# University-Community Partnerships to Develop a Mutually Beneficial Tool to Measure Age-Friendly Collaboration

**DOI:** 10.1093/geroni/igaa057.2447

**Published:** 2020-12-16

**Authors:** Althea Pestine-Stevens, Emily Greenfield

**Affiliations:** 1 Rutgers–New Brunswick, New Brunswick, New Jersey, United States; 2 Rutgers, The State University of New Jersey, New Brunswick, New Jersey, United States

## Abstract

Age-friendly community initiatives (AFCIs) are frequently described as community collaborations; the AARP program model encourages diverse stakeholder engagement to achieve its aims of improving the social and built environments for long lives. However, little is known empirically about how AFCIs function as collaborations and how these relationships and activities lead to community changes. This paper presents how we developed a social network analysis tool to measure collaboration in AFCIs, which emerged from multi-year, university-community partnerships on AFCIs in western New York and northern New Jersey. Iterative processes, including inductive analysis of qualitative interviews and facilitated meetings with local AFCI work-groups, provided opportunities to create survey items on collaboration with meaning specific to AFCIs. We describe this tool’s application as part of a survey of AFCI stakeholders, demonstrating how findings contribute both to advancing knowledge on AFCIs in general and directly informing the efforts of AFCI actors on the ground.

